# Editorials

**Published:** 1898-12

**Authors:** 


### EDITORIAL.

### 1898-1899.

WITH this number we close the nineteenth volume of the JOURNAL. The year 1898 will be remembered by the veterinary profession as the one closing the long period of depression in animal husbandry and the era of the lowest equine values in many years. The continued depression for more than four years reached its severest limit in 1898, and likewise the long-promised recovery from this period of great losses made itself felt throughout our entire land. The rapid decline of breeding for more than five years; the many times multiplied foreign markets; the increased needs at home from a war demand; the realization that the automobile is yet only a toy; the decline of the bicycle, and the increased demand for saddle-horses; the readjustment of business inequalities over our land, have all played a part in this steadily improving condition among those engaged in the horse-industry. The greater attention to pure food-products; the urgent need of sanitary police systems in every community; the wider foreign market for our surplus food-products, and the determination to hold these fields of consumers, have all added to the tide of prosperity flowing toward the fields in which the broadest and deepest interests of the veterinarian lie. With this better condition have come evidences of stronger support and still brighter prospects for veterinary journalism. During the year just closing the JOURNAL has been enabled to spend more than a thousand dollars in sending forth among the more than nine thousand veterinarians of North America special editions of the JOURNAL, and this contribution on our part has added to the JOURNAL the largest number of readers, the largest paid-up subscription list it has ever enjoyed during the past ten years, and though we have conducted since December, 1896, only a paid-in-advance list of subscribers, the JOURNAL has touched in the year just closing the largest subscription list ever accorded it. This has enabled us to give more freely to the profession, add new departments to our columns, and widen the field of information in every direction. With the same generous support in 1899 we shall maintain every advantage gained; and, with the best and largest corps of contributors in its history, we will keep ever foremost the aim we have kept as our guide—to "Lead Veterinary Journalism in America."

### OUR NEW DEPARTMENTS.

During the year just closing, the Journal, in addition to the widest range of general information, the assistance of the ablest writers it has ever enjoyed, has added to its columns a department of therapeutics, and later one upon surgery. These subjects, of so much practical interest to the everyday practitioner, will be maintained under the special direction of members of the profession well-known to the readers of the Journal and in every way peculiarly equipped for this work. The many advantages to be derived by one of the largest surgical practices in this country will find expression in these monthly contributions to our pages, while the field of surgery in city and country practice will from time to time be specially referred to by those who will conduct this department in the interest of our readers. The broad field of therapeutics will be monthly scanned for the freshest notes in applied medicine, and the newer drugs and remedies will be constantly recorded, that the profession may be kept in touch with the best thought and progress in these directions. Every assistance to those conducting these departments will be fully appreciated by the editorial direction and management and equally so by the largest number of readers ever enjoyed by the Journal.

---

### NEW YORK WINS FOR 1899.

The thirty-sixth annual meeting of the American Veterinary Medical Association will convene in New York City in September. This decision of the Executive Committee reaches us just as the last forms of the Journal are closing up, and the succeeding issue of the Journal will more fully report the decision arrived at by the vote of the Executive Committee.

We shall only say at this time that the sentiment seemed among the profession to lean toward this great metropolis. Those of the West who expressed the belief that New York was as far east as they could hope to go will feel well satisfied with this decision, while those of the New England States who favored Boston cannot find any fault with the decision on the score of geographical situation, and New York should have the strongest support from the New England States, as she will have of those in the territory east of the Allegheny Mountains, in making the meeting the grandest and best in the Association's history.

The Journal tenders its congratulations and pledges its most earnest assistance and support.

### ORGANIZE FOR BETTER VETERINARY LAWS.

MANY of the State veterinary organizations will this winter knock at the doors of their respective Legislatures for the enactment of laws looking to a proper recognition of the veterinary profession and its higher standing, and for the protection of the people from ignorant pretenders, quacks, and humbugs who prey like parasites upon a confiding public. It is unfair to say that intelligent communities or people should not allow themselves to be so imposed upon or taken advantage of; but when one thinks of how many directions one must trust to wise laws and ordinances for self-preservation, the public should not be left in this all-important direction without the strongest safeguards. One thought alone—how dependent upon the veterinary profession are our people, in the sanitary field and its adjunct of a nation's food-supply, over which the veterinarian is the only one fully equipped to shoulder its responsibilities and discharge them with fidelity to the people's interest—should awaken every good citizen to the safeguards needed to fill these places with competent and properly educated guardians.

Tennessee and Vermont have already planned for legislation in this direction. The former State has her veterinary organization well drilled. The latter Commonwealth, with her newly-born association, should alike command every assistance and support from the whole profession in its laudable undertakings, and every veterinarian should contribute such suggestions as will bring to these movements the support of an enlightened public, and thereby insure success. The opposition to all these movements invariably arises from ignorance and ignorant pretenders, who are only parasites on the profession's body and whose chief aim and purpose in life is that of deception and humbug, to the detriment of the progress, health, and happiness of every community.

